# Impact of COVID-19 in mental health trusts

**DOI:** 10.1177/13558196221116298

**Published:** 2022-08-08

**Authors:** Russell Mannion, Frederick H Konteh, Rowena Jacobs

**Affiliations:** 1 Professor of Health Systems, Health Services Management Centre, 1724 University of Birmingham, Birmingham, UK; 2 Research Fellow, Health Services Management Centre, 1724 University of Birmingham, Birmingham, UK; 3 Professor of Health Economics, Centre for Health Economics, 8748 University of York, Birmingham, UK

**Keywords:** COVID-19, mental health services, digital technology

## Abstract

**Objective:**

To explore how mental health trusts in England adapted and responded to the challenges posed by the COVID-19 pandemic, with the aim of identifying lessons that can be learned during and beyond the pandemic.

**Methods:**

Following a scoping study, we undertook 52 semi-structured interviews with senior managers, clinicians, patient representatives and commissioning staff across four case study sites. These sites varied in size, location and grading awarded by a national regulatory body. We explored how services have been repurposed and reorganized in response to the pandemic and the participants’ perceptions of the impact of these changes on quality of care and the wellbeing of staff.

**Results:**

Mental health trusts have shown great flexibility and resilience in rapidly implementing new models of care and developing creative digital solutions at speed. New collaborative arrangements have been stimulated by a shared sense of urgency and enabled by additional funding and a more permissive policy environment. But there has also been a significant negative impact on the wellbeing of staff, particularly those staff from a minority ethnic background. Also, there were concerns that digital technology could effectively disenfranchise some vulnerable groups and exacerbate existing health inequalities.

**Conclusions:**

Many of the service changes and digital innovations undertaken during the pandemic appear promising. Nevertheless, those changes need to be urgently and rigorously appraised to assure their effectiveness and to assess their impact on social exclusion and health inequalities.

## Introduction

The COVID-19 pandemic is testing the resilience of mental health services worldwide and has exposed the vulnerabilities of people living with mental ill-health. A report published by the World Health Organization during the first year of the pandemic highlighted that essential mental health provision had been severely disrupted in 93% of 130 of its member states.^1^ Reflecting the experience of previous respiratory viral epidemics – such as Ebola, SARS and influenza – many countries during the first year of the COVID-19 pandemic experienced a sharp rise in depressive, anxiety and stress disorders among the general population and a worsening of symptoms in people with pre-existing conditions.^2^

The proportion of adults in the UK reporting a clinically significant level of psychological distress has varied over the course of the pandemic, with an increase from 21% in 2019 to 30% in April 2020, which then dropped back to 21% by September 2020.^3^ More recent evidence suggests that there was a second deterioration in population mental health between October 2020 and February 2021 followed by a period of recovery.^3^ Declining population mental health has therefore coincided with periods of national lockdown, and presentation rates have fluctuated in line with lockdowns. This implies that containment measures designed to suppress the spread of the virus, including enforced social distancing and school closures, may have eroded the protective factors generally associated with good mental health (social connectedness and companionship, economic security, educational engagement and outdoor physical exercise) as well as exacerbating risk factors associated with poor psycho-social wellbeing (anxiety, fear, loneliness, domestic abuse and financial hardship).^3^

In October 2020, forecast modelling by the Centre for Mental Health charity predicted that an additional 500,000 people in England will require support for their mental health over the following 2 years as a direct consequence of the pandemic.^4^ At the same time, the mental health charity Mind declared that England was in the grip of a ‘mental health emergency’^5(p1)^ and called for more government investment in mental health services. The government unveiled a mental health recovery action plan in March 2021, which included £500 million of additional funding to help the National Health Service (NHS) deal with the expected surge in demand for mental health services.^6^

The current study was conducted within England’s NHS – a publicly funded, single-payer healthcare system providing universal coverage in which most services are provided free at the point of service. Most NHS hospital providers are run by trusts. There are 54 mental health trusts in England providing a wide range of services, either delivered on a standalone basis or in partnership with other sectors (acute, ambulance, community trusts), other agencies (housing, police, prison services), or other organizations (especially voluntary/social enterprises). Most mental health services are for those who live in the local area, although some mental health trusts may accept national referrals. Care is generally delivered across three settings: care provided in the community (where a service user accesses services from home), inpatient care (usually an inpatient ward) and secure care (a locked setting). Access to secondary mental health services is usually arranged through the patient’s primary care medical doctor or sometimes via self-referral, the criminal justice system or – in the case of children and young people – schools and colleges.

The pandemic emerged when mental health services in England were already overstretched with severe workforce shortages and struggling to meet existing demand.^7^ Our study aimed to understand how mental health trusts in England adapted and repurposed services to cope with the COVID-19 pandemic, and what lessons can be learnt from this.

## Methods

Our research was based on interviews we conducted on the impact of COVID-19 from the beginning of the pandemic in February 2020 up to the date of each interview. The study comprised two sequential stages:

### Scoping phase

To ensure our research was grounded in the latest national policy developments and service priorities, we first undertook a scoping study. We interviewed six key national informants with knowledge of national mental health policy, comprising two representatives from NHS England, and one representative each from the Care Quality Commission (CQC, the independent regulator of health care in England), the Mental Health Commissioners Network (a national initiative aimed at providing collective voice and representation for organizations commissioning mental health services), the Healthcare Financial Management Association (which supports financial managers in the health sector), and the *Get it Right First Time* programme (a national scheme that uses clinically led investigations to improve patient care and treatment). The interviews were undertaken between March and April 2021.

We also reviewed relevant documents and statistical material produced by government agencies, health think tanks, regulators, patient representative organizations and mental health charities. The information derived from the scoping phase was used to inform the design of the case studies, most notably the focus of the research questions and themes explored in the case studies.

### Case studies phase

We utilized a comparative case study design conducted in four mental health trusts in England. This afforded the required degree of variation while remaining feasible within the study constraints. The case study sites represent a variety of contextual factors, including mental health services delivered, size, geographical location and the latest performance rating by the CQC. To protect anonymity, we identify the sites using the names of Cumbrian mountains in England. The sites were:

*Blencathra* provides the full range of mental health services, as well as support for people with learning disabilities. It employs over 2400 staff. It is based in the East Midlands area of England in a rural setting and serves a population of more than 700,000 people. In 2020, the trust was rated by the CQC as overall Good.

*Helvellyn* provides a range of community, mental health and acute hospital services. It employs over 4000 staff. It is based in the south-west of England in a mostly rural area with no large urban centre and serves a population of more than 500,000 people. In 2019, the CQC rated the trust as overall Good.

*Scafell* provides a range of integrated mental health and social care services to people of all ages. It employs more than 2000 staff. It is based in a major city in the south of England and serves a population of over 1.2 million people. It serves the largest population yet has the lowest staff size of the four case studies. In 2019, the CQC rated the trust as overall Good.

*Skiddaw* provides specialist mental health, learning disability and community health services. It employs over 3500 staff. It is based in the south of England and provides services across a mixed urban/rural setting with a population of more than 900,000 people. It recently received a national award for being an advanced digital organization. In 2020, the CQC rated the trust as overall Outstanding.

We undertook 52 semi-structured interviews across the case study sites. Participants were purposefully selected to include senior managers and clinicians with a knowledge of strategic service planning, as well as patient representatives. Those interviewed were: the trusts’ four chief executives, 16 medical/clinical directors, three directors of nursing, 15 board directors and service managers with a range of organizational roles and responsibilities, four consultant psychiatrists and four patient representatives (one from each trust). To provide an external perspective, we also interviewed six senior managers from local Clinical Commissioning Groups, organizations that commission services from the trusts.

A topic guide based on information generated from the scoping phase was later developed and adapted during data collection in the case studies to further explore new issues and emerging categories.

The interviews took place between March and December 2021. The date of the interview is provided alongside each illustrative quote in the Results section. The interviews were conducted using online video conferencing software and lasted between 35 and 45 min. With the consent of participants, interviews were digitally recorded and professionally transcribed verbatim. The transcripts were supplemented with field notes.

Qualitative coding software (NVivo) was used to facilitate data storage and retrieval in analysis. The five stages of the Framework method (familiarization, theme identification, indexing, charting and interpretation) were followed, and this structured the analysis of data.^8^ In order to improve the validity of the study, where possible, we cross-referenced accounts from individuals and triangulated the evidence emanating from different sources, including internal documents (e.g. clinical governance reports) and external reports. We also audited the various sources of data to search for negative or disconfirming evidence that contradicted or was inconsistent with the emerging analysis.

Our analysis is based on the perceptions and subjective experience of individuals. But while there is a patterning of experience which is unique to each case study site, our analysis extends those experiences by integrating and drawing out the common themes across the four sites. Quotes are used to illustrate each theme and labelled with the professional group of the interviewee and the date of interview. The quotes have been edited for language and flow.

## Results

Our findings are structured around six key policy and management-related themes, which were generated during the scoping phase and used to inform the research questions explored in the case studies.

### Dramatic changes in demand for and access to services

The participating trusts have witnessed significant changes in the pattern of referrals over the course of the pandemic, with all experiencing a significant drop in demand for services during the first wave (February to March 2020, with a national lockdown running from March to June 2020). A common view across the case studies was that many people were avoiding accessing mental health services during the first wave because of concerns about infection or because of concerns that they did not want to burden the health system at a time of crisis. There was also a view that many people thought that mental health services were not ‘open for business’.

During the first lockdown we saw a significant decrease in the number of referrals coming through into services. We have certainly had to do an awful lot of reminding people, GPs, the public, that we hadn’t gone anywhere, and that mental health services were continuing to work – you know, continuing to be open. (Associate director of operations, Blencathra, 28 June)

Towards the end of the first lockdown we were getting more people with severe anxiety referred so that would be people that we wouldn’t have seen before. (Consultant psychiatrist, older people, Scafell, 1 July)

Trusts saw an unprecedented surge in demand for services once lockdowns were lifted and social restrictions relaxed. A common pathway into mental health services prior to the pandemic, for children and young people in particular, was via schools. With school closures during the first half of 2020, this pathway was disrupted. Consequently, referrals to children and adolescent mental health services fluctuated in line with schools being closed and reopened.

There’s been additional demand for child and adolescent mental health services, and the government has provided some additional investment that we have been able to use to respond to that. (Patient representative, Blencathra, 22 December)

Increasing pressure on services was not only due to an increase in referrals but also to the acuity of people presenting with mental health conditions, with a common refrain being that patients were presenting ‘sicker’ or more ‘severely ill’. All four trusts saw an increase in self-referrals and an upsurge in the number of people presenting during a mental health crisis, as well as a sharp rise in referrals for people with no previous history of mental illness.

What we have seen is the acuity of patients, of children and young people, has increased. So, by the time they’re getting to us, they are more unwell. (Clinical Commissioning Group senior manager, Helvellyn, 9 August).

Everybody from children through to older adults will have had a lack of social integration and socialisation - that sort of thing is bound to have a mental effect. (Patient representative, Skiddaw, 8 August)

Child and adolescent mental health services, eating disorders, autism and learning disabilities services all experienced a sharp rise in referrals across all four trusts. But other increases in demand and activity were more locally specific. For example, in Blencathra there had been an upsurge in demand for inpatient wards and personality disorders services whilst demand for home treatment teams and community mental health services had increased in Helvellyn. Conversely, some services had seen little increase in demand activity over the course of the pandemic. For example, Improving Access to Psychological Therapy (IAPT) services had not experienced the expected surge in demand predicted at the start of the pandemic in any of the case study sites. Despite these variations in demand, all the participating trusts reported that they managed to deliver a full range of essential mental health services during the pandemic.

### Delivery of care reorganized

Each trust made a series of radical transformations in how services were organized and delivered, with these transformations occurring at a speed and scale previously unseen. In partnership with community services, the immediate response was to follow national guidance with regard to accelerating the discharge of medically fit patients to make room for COVID-19 patients, with many acute mental hospital wards reorganized to ensure physical separation between COVID-19 positive and COVID-19–free patients.

Trusts reported having to close down specific wards either due to a COVID-19 breakout or because of serious staff shortages (due to staff self-isolating).

We had outbreaks of COVID within the wards. It meant that you had to close down wards or have sort of red wards, so you couldn’t admit. So, we had the bed capacity but we had issues getting people into beds because there were very few wards that you could admit to. (Clinical manager home treatment teams, Scafell, 23 July)

Scafell and Skiddaw reported that out-of-area placement of patients increased, particularly during the first phase of the pandemic, due to pressures on bed capacity.

Over the last sort of couple of years we’d done really well at reducing our out-of-area admissions, our use of independent sector beds…[But] we now have no option but to use independent sector beds and to place people in placements that are not in the local area. So it’s a really pressured bed position for both children and young people and adults. (Director of strategy, Scafell, 14 May)

Urgent 24/7 mental health telephone helplines were introduced, and services providing individuals in distress with timely support in the community were expanded.

There was a perception that many of these rapid changes in organization and delivery were facilitated by loosened hierarchical and bureaucratic constraints. This increased local provider autonomy and empowered local managers and healthcare professionals to implement solutions at speed.

### Increased use of digital tools and online communication systems

All four trusts accelerated the roll out of new models of care supported by mobile and digital technologies, most notably using digital tools to facilitate remote patient appointments. These changes helped improve service efficiency by reducing the need for staff to travel, which left more time for other work, as well as reducing the trusts’ carbon footprint (which contributed towards meeting the government’s commitment to achieving a net zero NHS by 2045).^9^

Due to social distancing requirements restricting the number of people allowed in a room, internal staff meetings were quickly switched to video conferences. Staff were also quickly trained in the use of new information technology, allowing them to conduct remote assessments of patients using a range of online platforms such as Zoom and Microsoft Teams. Patients were regarded as having benefitted because services were more responsive and immediate. Some clinical areas – such as IAPT – adapted very quickly to remote service provision because these services had started to use more digital technology before the pandemic.

Scafell, Skiddaw and, to a lesser extent, Blencathra were already digitally enabled and poised to scale up the use of digital technology fast. Skiddaw had recently received a national award recognizing it was a leader in digital technology and was developing a range of innovative organization-wide digitally enabled systems, including paperless wards and services, digitizing observations and real time data on bed capacity dashboards for the crisis team.

We’re really fortunate to be quite a digitally enabled organisation, so we already had numbers of virtual platforms…We were able to move, I would say, probably more swiftly than some others to a virtual platform. So, some of our staff were saying they were able to make a fairly seamless transition - they just moved their contacts online. (Director of nursing, Skiddaw, 16 June)

From an IAPT perspective, prior to the pandemic, we were doing lots of remote working anyway, as a large rural county, where we’ve already experimented a little bit with video conferencing and with telephone work and had been quite actively involved in looking at those options. So, when the pandemic hit, it meant that we just really upscaled very quickly the digital options. (Clinical manager, Blencathra, 6 April)

In contrast, Helvellyn was less well prepared. For example, many of its clinicians did not have a personal laptop computer prior to the pandemic.

Suddenly there was need for everybody, all clinicians to have a laptop….we weren’t prepared for that and we were on the back foot. (Medical director, Helvellyn, 19 July)

In spite of the largely positive views on the increased use of technology, a number of negative aspects were highlighted in the interviews. First, a widely held view was that the increased use of digital technology could disenfranchise some groups and exacerbate existing health inequalities. For example, it was felt that many older people may not be familiar with the use of digital technology and those on lower incomes may not be able to afford to buy devices, such as smartphones, or pay for reliable broadband access. In some cases, the trusts provided digital equipment, tablets and phones to service users to ensure that they were not excluded from services.

We also supplied equipment to those patients that had high levels of digital poverty, so didn’t have a phone, didn’t have any way of accessing this kind of technology. So, we did provide those as well, those platforms for patients. (Regional director, Helvellyn, 2 June)

Second, online and remote service delivery was not felt to be appropriate for those requiring a private ‘safe space’ or refuge to talk about personal and confidential issues without the risk of being overheard. Examples cited included women who are exposed to domestic violence, children experiencing neglect and people not confident in sharing details of their sexuality with their families.

If you’ve got a situation where there’s domestic abuse in a household, you’re not going to be able to engage with that person in an open way unless they’ve got a safe space to talk from. So, there were a number of groups which we were quite conscious of and have tried to create alternative plans for. So, we continued to offer face-to-face in certain circumstances. (Clinical manager, Blencathra, 6 April)

Third, remote consultation was considered unsuitable for certain mental health conditions, such as people with autism. Finally, there was a strong view among staff that services delivered should reflect individual choice and a belief that some patients and service users strongly prefer in-person consultations rather than online appointments.

### Variation in the quality of services

The trusts all reported that it was hard to determine the precise impact of the pandemic on the quality of services provided, as the collection of many routine quality measures and indicators had been suspended during the peak of the pandemic.

We don’t know the true quality benefits of digital working yet because we’ve got no objective measures at the moment to really understand that. (Director of operations, Blencathra, 29 June)

Where providers have managed to elicit patients’ feedback it was reported that this was generally very positive.

However, across the four trusts, staff expressed nuanced views about the impact of the pandemic on the quality of services they had been able to deliver. In fact, the perceived impact on quality differed between staff within the same organization. For example, the director of operations for older people in Blencathra gave a positive assessment of service quality (their own service in particular) while the medical director in the same organization described how the overall quality of services had been adversely affected by the pandemic.

However, there were widespread concerns about the quality of service that could be provided through remote assessment for groups who are more likely to benefit from in-person assessment – for example, those with personality disorders.

There’s also a question about the quality of the assessment that you can make for someone through a virtual platform consistently because you can’t beat on some occasions going into someone’s house and seeing what’s happening, and how they connect, and how they relate. I think, for example, some of the personality disorder services - if you’re paranoid and schizophrenic, and worried about computers, your experience is going to be very different (Chief executive, Scafell, 14 May)

### Negative impact on staff safety and wellbeing

Dealing with the pressures associated with the pandemic had a significant negative impact on the health and wellbeing of staff. This was especially true for frontline clinical staff in all the trusts, with staff feeling ‘burned out’, tired and exhausted.

I would say the number one challenge for me and my team was fatigue. We were working eight, 10, 12 h a day into the evening, certainly at the beginning of the pandemic because it was all new, we didn’t know what was coming. So everyone was very tired and everyone was giving everything to the cause. (Director of informatics, Blencathra, 1 April)

Staff morale was adversely affected, due to some people being away from their workplace for a long period and the consequent lack of in-person interaction with colleagues.

Staff were also worried about their personal safety due to a lack of personal protective equipment, particularly during the first wave of the pandemic, as well as being concerned about taking the virus home and infecting those they lived with.

Wearing a mask at work all day does have an impact. Being frightened for your own wellbeing and frightened about going back to your family, in case you give them COVID, has a massive impact. (Chief executive, Scafell, 14 May)

Staff from minority ethnic groups were reported to be particularly concerned about being infected with COVID-19 and passing it to family members. This was especially the case at Blencathra, Scafell and Skiddaw, all of which have a very diverse workforce. Such staff were also concerned about the lack of opportunity to take a break from work and visit their families living abroad. Staff members with physical disabilities were also reported to be particularly fearful about the risks of infection.

If you were in an ethnic group where you kept being told you were more at risk…that is going to have an impact on you…I think, equally, as well as ethnicity we had those people with a disability, who were really worried. So, there were absolutely pockets of people that were more concerned than others. (Director of nursing, Skiddaw, 16 June)

To support staff wellbeing, each trust implemented a suite of psychological, emotional and wellbeing support services, including online digital resources, group sessions and talking therapies. Blencathra set up a dedicated wellbeing service for minority ethnic staff, as the organization recognized that they were most at risk and required targeted support and resources.

We’ve put in place fortnightly BAME [Black, Asian and Minority Ethnic] and Allies meetings. So, every fortnight all of our staff who identify in that group have a support mechanism where they can bring their stories, share their information about the impact of COVID on them, and do that with executive directors in the room. (Director of strategy, Blencathra, 7 April)

### System-wide collaboration accelerated

The general view from the case studies was that the necessity of responding to challenges posed by the pandemic accelerated progress towards the development of more integrated care systems and facilitated better joint collaborative and working arrangements across organizational boundaries. This was reported to be due to trusts, commissioning organizations and partners recognizing that they were ‘all in this together’ and needed to work together to address the same challenges. This allowed organizations to look beyond their immediate self-interest, which had previously (pre-pandemic) hampered effective collaboration.

There was that sense of, ‘We’re all in this crisis together and we’ve all got to do what’s best for the outcomes for people’…For years, people have worked with the theoretical notion of putting aside their loyalties to their own organisations, well, this kind of made it happen. So, I think it accelerated those relationships and that system working exponentially. (Associate director, Blencathra, 12 May)

A key factor associated with improved system relationships was the positive effects of increased funding and new care arrangements, by which trusts were allocated a block contract, which removed the need to negotiate separate individual service-level contracts. This latter factor served to mitigate some historical organizational conflicts and ‘turf wars’ between local providers, as they did not need to compete with each other for funds.

The boundaries always existed because we found it difficult because of commissioning arrangements. Those boundaries have been knocked down somewhat, we’ve found a way to work around them. I suppose the hope is that that continues rather than once we go back to business as usual, we don’t all put our boundaries back up and stop helping each other because that’s not helpful for anybody. (Regional director, Skiddaw, 2 June)

But challenges remain. In Helvellyn, for instance, it was noted that there still existed some tensions within primary care and that relationship-building was required to improve collaborative working. Further, as the worst of the pandemic recedes, old ways can re-emerge.

People were just working for a common need, with one objective. A lot of those organisational tensions disappeared. But they are quickly back in now. But at the height of [the pandemic] it was incredible. (Clinical Commissioning Group senior manager, Skiddaw, 24 November)

## Discussion

The pandemic has posed unique challenges for mental health services in England due to reduced service capacity at a time of fluctuating demand. As our case studies demonstrate, the pandemic has acted as a significant catalyst for major service innovations and opened up new pathways of care in areas that over many years had made only incremental progress. Mental health trusts have demonstrated a high degree of flexibility and resilience by quickly transforming the way in which services are organized and delivered.

In particular, the rapid deployment of digital technology and the shift to remote provision has played a vital role in connecting providers with service users, and allowed healthcare professionals and teams to maintain links across health and care systems. New collaborative arrangements have been stimulated by a sudden shared sense of urgency and enabled by additional funding, a more permissive policy environment and lighter-touch regulation.

Although they differed in terms of ‘digital maturity’, all four case studies were enthusiastic adopters of new technology and reported a range of positive experiences. But alongside these benefits came a number of challenges which would need to be overcome if the digital transformation of services is to achieve the desired improvements in service delivery. First, although digitally enabled remote care was often the only option available to service users during the first stages of the pandemic and subsequent lockdowns, it is not necessarily the case that many patients and users would prefer to continue to access services in this way. Thus, the need for providers to be responsive to patients’ choice – offering face-to-face service as needed – remains a key consideration. It is also important to explore service users’ and staff perspectives and experiences of digital technology and remote consultations, to appreciate the impact this way of working has had on patient safety and quality of care.^12–13^ Our case studies also noted concerns that a ‘digital divide’ in access to technology may be exacerbating existing health inequalities and socially excluding already vulnerable groups. This highlights the importance of retaining alternatives to digital services as well as the need to provide suitable digital resources for those unable to afford them.

It is clear that staff working in mental health services have risen to the demands of the pandemic, but have carried a heavy burden with significant personal costs for their own physical health (exhaustion, fatigue) and mental wellbeing (stress, anxiety, morale), particularly for those working in frontline clinical services. These issues align with the findings of international studies on the impact of the pandemic on the physical and mental wellbeing of healthcare staff.^14–16^ It is encouraging that all the participating trusts were implementing a range of wellbeing services to support staff during the pandemic. However, it will be important to ensure that appropriate emotional and wellbeing services are sustained in the aftermath of the pandemic, when demand is likely to increase, particularly given the expected rise in the demand for long-term treatment for post-traumatic stress disorders.^17^

As part of ongoing efforts, attention should also focus on developing supportive organizational cultures where staff feel able to speak up about problems or concerns, they have for themselves, as well as for patients.^18^ And organizations must develop appropriate support when staff identify sources of workplace strain.^19^ In assessing what works best in supporting the wellbeing of staff, it will be important to take into account a diversity of staff views, not least the perspectives and experiences of those from minority ethnic groups who have been at increased risk and have been disproportionally affected by the pandemic.^19^

While the pandemic has led to radical changes in the way mental health services are delivered and used, careful appraisal is required to produce rigorous and relevant evidence on what has worked (how, why and for whom) and what needs retaining, modifying or abandoning before service changes are embedded and become the ‘new normal’. Research and evaluation in this area will need to exhibit a number of features. First, the pace of change and pressures of managing the pandemic has meant that major service shifts happened with little involvement from patients and the public. Approaches to research and evaluation that engage with and take heed of the voices of diverse service users – particularly those with lived experience of mental illness, as well as frontline staff – will be crucial to identifying service changes worth retaining post-pandemic.

Second, evaluation studies would benefit from adopting a ‘rapid evaluation’ approach, to enable timely findings to support the urgent spread of successful innovation more widely.^10^

Finally, the changes are not only structural and procedural, but also cultural and behavioural. Service shifts seen during the pandemic have upended many of the traditional assumptions, beliefs and working practices that have been affirmed over decades and woven into the fabric of mental healthcare delivery.^20^ Any future evaluations should seek to identify those positive values and behaviours, which have been suppressed during the pandemic and which might need to be reinforced; those that have newly emerged and are facilitative of high performance; and those that are damaging to patient care. Of particular concern is the need to be alert to the role of local professional subcultures which, at different times, may be driving forces for change, defenders of the status quo (for good or ill) or covert counter-cultures quietly undermining necessary change.^11^

## Limitations

There are four main limitations in our study. The first relates to the generalizability of the findings beyond the four case study sites. Although the case studies were sampled purposefully to reflect a range of organizational characteristics, as well as being dispersed geographically across the country, we cannot state categorically that our findings are necessarily generalizable to all mental health trusts in England. However, set alongside interviews with national stakeholders and background statistical data, we believe that our study has uncovered some important aspects of the impact of COVID-19 that are transferable to mental health trusts more generally.

The second limitation is that the study only represents a snapshot up to December 2021. The full chronology of the impact of the pandemic on mental health trusts remains an ongoing process.

The third limitation may be its focus on the perceptions of a small number of senior managers. We were unable to triangulate their perspectives and experiences with those of staff lower down the organizational hierarchy and therefore we were unable to fully capture frontline perspectives. However, the benefit of focusing on senior staff is that they sit at the apex of organizations and have a strategic overview of how services are being affected. We also interviewed managers from local commissioning groups to obtain an external perspective.

The fourth limitation is that we only interviewed a small number of patient representatives. As such, we were unable to fully capture the patient perspective.

## Conclusions

The pandemic has presented a huge challenge for mental health providers and for those living with mental illness. The results in our case studies are testimony to the way in which providers have stepped up to the task and shown great flexibility and resilience in responding to the vast array of challenges wrought by the pandemic. Yet the mental health workforce has been severely overstretched with a consequent significant negative impact on the wellbeing of staff, particularly for those from minority ethnic backgrounds, who have been disproportionately affected.

Although many of the initial service changes appear to be promising, amid growing waiting lists there is an urgent need for ongoing rapid appraisal to reassure of their cost-effectiveness, sustainability and impact on health inequalities. The mental health toll of the pandemic will play out in the years, if not decades, to come and a full assessment of its repercussions on population mental health and the demand for services remains an ongoing task.
